# Economic distress and perceptions of sexual intimacy in remarriage

**DOI:** 10.3389/fpsyg.2022.1056180

**Published:** 2023-01-06

**Authors:** Joshua J. Turner, J. Scott Crapo, Olena Kopystynska, Kay Bradford, Brian J. Higginbotham

**Affiliations:** ^1^Department of Human Development and Family Studies, Utah State University, Logan, UT, United States; ^2^Department of Family Life and Human Development, Southern Utah University, Cedar City, UT, United States

**Keywords:** remarriage, economic distress, relationship functioning, relationship satisfaction, multidimensional family development theory, sexual intimacy

## Abstract

**Introduction:**

Economic distress and the relationship stability of remarried couples has been subject to some exploration, but less emphasis has been placed on how economic distress among remarried couples impacts other relationship domains, particularly sexual intimacy.

**Methods:**

Through the lens of multidimensional family development theory (MFDT), this study utilizes longitudinal data over a three-year period to examine the links between economic distress, couple engagement, relationship satisfaction, and perceptions of sexual intimacy among remarried couples (*n* = 1,161 couples; 97% White).

**Results:**

Through a dyadic structural equation model, results showed that wives’ report of economic distress was directly related to their self-rejection of a partner’s sexual advances. Findings also revealed gender differences in how both relationship satisfaction and couple engagement influenced one to accept or reject their partner’s sexual advances, with couple engagement acting as a significant predictor for wives. Relationship satisfaction was also found to explain (i.e., mediate) the relation between economic distress and sexual intimacy, but only for husbands.

**Discussion:**

Implications for further research and interventions designed to strengthen the relationships of remarried couples dealing with economic distress and intimacy issues are offered.

## Introduction

Remarriage has become commonplace in United States culture (Livingston, 2014). Remarried couples often face additional challenges compared to couples in first-order marriages (DeLongis and Zwicker, 2017), including economic distress (e.g., disrupted wealth accumulation or financial obligations tied to a previous relationship; Falke and Larson, 2007; Ganong and Coleman, 2017; Kapelle and Baxter, 2021) and prior relationship dissolution (e.g., divorce; Snyder et al., 2016). Also, considering that many remarried couples bring children from previous relationships, they face additional stressors of forming a stepfamily (DeLongis and Zwicker, 2017). Such topics have been at the forefront of the research efforts focusing on remarriage.

In this study, we examine the extent to which economic distress among remarried couples impacts other relationship domains, particularly the perception of sexual intimacy, a topic that has been less researched in the field of family science (Crapo et al., 2021). Advancing research on the factors that impact sexual intimacy could help identify ways to improve relationship stability for this population, which has been found to be vulnerable to relationship dissolution (Copen et al., 2012). Indeed, past research with general populations has found that higher satisfaction and greater frequency of sexual intimacy is positively related to higher levels of relationship satisfaction and commitment (Dobson et al., 2020; Dobson and Ogolsky, 2022). Conversely, lower levels of sexual satisfaction and frequency has been linked to lower levels of relationship satisfaction (Kim et al., 2018). The findings of the current study, augmented with a theoretical framework, may help researchers and practitioners better understand the relationship functioning of remarried couples, and inform interventions to strengthen the relationships of remarried couples dealing with economic distress and sexual intimacy issues.

### Theoretical framework: Multidimensional family development theory

Multidimensional family development theory (MFDT; Crapo and Bradford, 2021) is a theoretical framework that reconceptualizes traditional family development theory by explaining family development as a process that occurs concurrently within four different dimensions: (a) personal (e.g., growth, biological maturation), (b) vocational (e.g., socioeconomic status), (c) couple (e.g., relationship functioning, sexual intimacy), and (d) generative (e.g., parenting, community engagement). In applying MFDT to the intimacy issues of remarried couples, the couple dimension is the focus, especially with its attention to pairing and a couple’s sexual and emotional development (Sassler, 2010). But, by also emphasizing the role of economic distress, relationship satisfaction, and couple engagement, the personal (i.e., sexual and socioemotional development) and vocational (i.e., the abilities needed to provide for oneself and one’s family members) dimensions of MFDT also come into play. MFDT posits that changes in one of the other dimensions can impact the trajectory of the couple dimension (Crapo and Bradford, 2021). That is, each of the dimensions of development influences, and is influenced by, the other three dimensions, within a person and across a family. The nature of this influence is complicated; in short, as developmental events shift trajectories (the potential of future events occurring) within a dimension of development, they (may) simultaneously shift the trajectory of other dimensions of development.

Perhaps one of the more intuitive ways to think about this interrelation of dimensions is through the concept of alignment and misalignment, which is defined as “the ways in which the ordering and interaction of events in the individual dimensions of development increases or decreases a family’s ability to meet their family developmental tasks” (Crapo and Bradford, 2021, p. 10). This family developmental task is the responsibility of a family to meet all of the developmental needs that exist in each dimension of each person. As such, the developmental event of remarriage in the couple dimension may be misaligned with the vocational dimension if the couple is not in a position to support the complexity of a remarried family. In this specific instance, misalignment between the vocational dimension and the rest of the multidimensional and family developmental space may be manifested through economic distress. Misalignment makes it more difficult to meet developmental needs, and so the needs in the couple dimension are likely to go unfulfilled, reducing the likelihood that the remarried couple will be able to develop a mutually satisfying sexual relationship, which is a vital component of the trajectory of couple development.

What is more, remarriages often have a unique set of needs resulting from a specific developmental history. That is, previous developmental events (i.e., marriage and divorce), in conjunction with United States legal and social contexts, generate additional needs within many remarriages, many of which are often conflicting in nature. These can include demands in the vocational dimension (i.e., child support obligations and former partners) that may conflict with attempts to nurture a new romantic relationship in the couple dimension (Papernow, 2013; DeLongis and Zwicker, 2017). As a result, the resources and capacities spent on additional, complex needs that exist outside of the couple dimension may increase misalignment between the vocational dimension (as manifested by economic distress) and the couple dimension, which is the focus of our current study.

## Literature review

### Economic distress and sexual intimacy in marriage

There is empirical evidence of the effect of misalignment between the vocational dimension and the couple dimension. Economic distress is one of the strongest predictors of conflict and perceptions of relationship satisfaction within marriage (Britt and Huston, 2012; Wheeler and Kerpelman, 2016; Leavitt et al., 2019). Left unresolved, economic distress can lead to negative spousal interactions (Dew and Dakin, 2011), feelings of unfairness (Jenkins et al., 2002), and higher levels of family disorganization (Patterson, 2002). Research has also linked economic distress to sexual intimacy issues (Dew et al., 2012) and lower sexual satisfaction (Hill et al., 2017).

Recent research on this topic (Saxey et al., 2021; Wikle et al., 2021) suggests that although perceived economic distress can have a detrimental impact on sexual satisfaction, these problems can possibly be alleviated by effective communication about finances and financial therapy interventions. Specifically, Wikle et al. (2021) found that communicating about finances assisted couples in adapting to financial stress and especially helped wives avoid negative sexual consequences. Meanwhile, the work of Saxey et al. (2021) found that wives’ financial management behavior had an indirect, negative impact on both her and her husband’s sexual satisfaction, with wives’ perceptions of economic pressure serving as a mediator. These latter findings point to examples of partner effects for couple sexual outcomes, while holding important implications for the role of financial therapy in early marriage, especially as it relates to constructive financial and sexual communication.

Remarried couples generally experience higher levels of economic distress than couples in first-order marriages, which can stem from disruptions in wealth accumulation brought on by divorce. This can prove difficult to recover from (Kapelle and Baxter, 2021), and create disparities in wealth stratification between divorced and continuously married individuals (Kapelle, 2022). Further, divorce can also bring financial obligations to ex-spouses and children from previous unions (Ganong and Coleman, 2017). Such financial commitments may give rise to resentment (Sweeney, 2010) and can impact how spouses interact with one another (Kelley et al., 2018). Indeed, Crapo et al. (2021) found that early financial stress was associated with perceived decreases in the frequency of sexual initiation among husbands in remarriage, but not for wives. Although the above research establishes a link between economic distress and relationships, it does not clearly or concisely explicate the nature of this connection. The propositions of MFDT highlight the interplay between the couple and vocational dimensions as it relates to balancing the developmental needs of each dimension. For instance, if access to resources and opportunities are lacking in the vocational dimension, economic distress may become more pronounced, thus impacting the dimension of couple development (Crapo and Bradford, 2021), in terms of the frequency of sexual intimacy. Such a situation is similar to the idea of stress spillover, in which external stress factors (such as finances) can negatively impact the resources a couple has to nurture their relationship or address interpersonal relationship issues (Buck and Neff, 2012). In the next section, we articulate more precisely the connection between economic distress and sexual intimacy in remarriages as proposed by MFDT.

### Economic distress as misalignment and the trajectory of couple development

**Figure 1:**
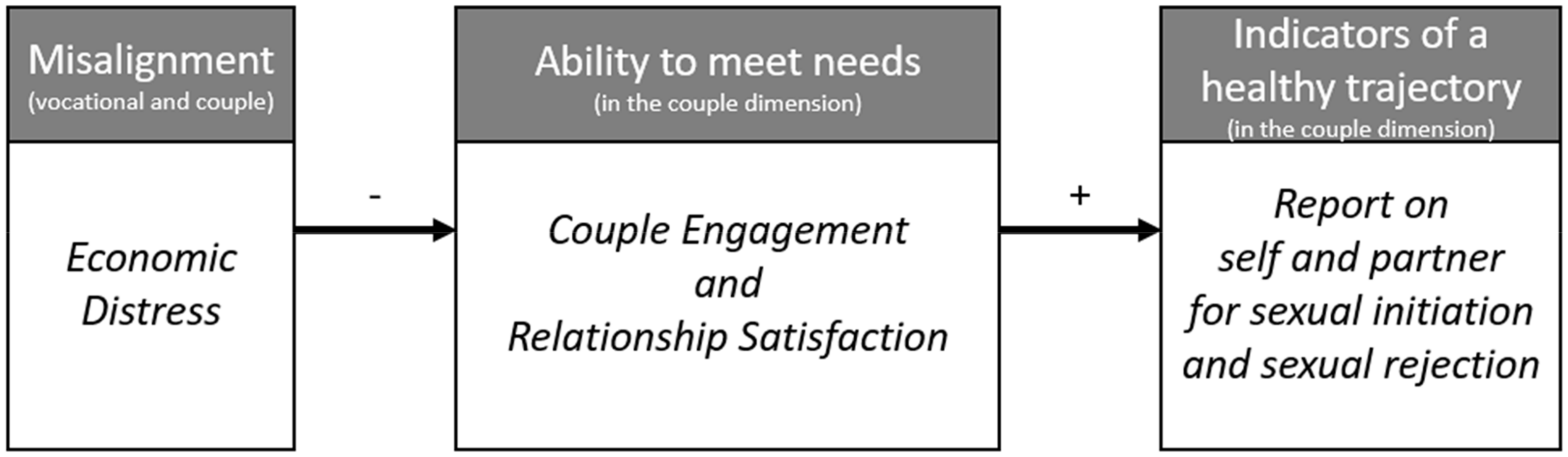
Related tenets of MFDT and their operationalization.

In defining economic distress as a measure of misalignment, the propositions of MFDT indicate that the reason economic distress affects the couple dimension in remarriages is because the couple would have greater difficulty in meeting their needs within the couple dimension (i.e., the portion of the family developmental task associated with development in the couple dimension). Although this could be conceptualized in many ways, we focus on two key indicators of needs being met: (a) the subjective evaluation, the success of the relationship at providing a satisfactory experience for the individual, or relationship satisfaction; and (b) the enacted behaviors, the observable manifestation of needs being met within the couple dimension, referred to as couple engagement. In MFDT, unmet needs mean than that the current trajectory is not pointing to the most optimal future events in that dimension. As such, an inability to meet needs as a result of misalignment would then result in greater likelihood of negative sexual events (such as rejecting or perceiving rejection of sexual advance), and a decreased likelihood of healthy sexual couple behaviors (such as initiating, or perceiving a spouse initiating, sexual intimacy) occurring as the couple dimension develops in a suboptimal way. See Figure 1 for a visual representation of this theoretically-derived mediated mechanism.

#### Relationship satisfaction and sexual intimacy

One measure of the subjective perception of relationship is relationship satisfaction. Substantial research has examined the associations between relationship satisfaction and sexual intimacy. For instance, earlier studies found that sexual satisfaction was related to marital well-being and satisfaction (Henderson-King and Veroff, 1994) and lower levels of marital instability (Yeh et al., 2006). More recent research largely supports these findings, including the work of Dobson and Ogolsky (2022) and Dobson et al. (2020), which found that greater sexual satisfaction and frequency of sexual intimacy was linked to higher levels of relationship satisfaction. In contrast to these findings, low levels of sexual satisfaction and frequency have been found to adversely affect relationship satisfaction (Kim et al., 2018).

Recent longitudinal research has been instrumental in examining the directional relationship between relationship and sexual satisfaction, with varying results. For instance, Vowels and Mark (2020) found that relationship satisfaction was a better predictor for sexual satisfaction than vice versa, contradicting the idea of a bidirectional relationship that has often been proposed between these two variables (McNulty et al., 2016). Still, other longitudinal research by Cao et al. (2019) found that the direction of this relationship can vary by gender, with the early sexual satisfaction of husbands predicting their marital satisfaction later on and the early marital satisfaction of wives predicting future sexual satisfaction. The mixed results of these studies makes it necessary to conduct more research in this area.

#### Couple engagement and sexual intimacy

In addition to the subjective view, the performed behaviors of the couple also can serve as a measure of how well needs within the relationship are being met. Healthy day-to-day couple engagement (e.g., effective communication and egalitarian approaches to household labor) has been associated with greater relationship satisfaction and more satisfying sexual intimacy (Olson et al., 2021). Although the activities in which couples may engage in varies, we focus on indicators that have been associated with sexual intimacy: shared household labor, leisure activities, and communication. Shared household labor is one of the most fundamental aspects of couple engagement and is also a major source of relationship satisfaction (Ciciolla and Luthar, 2019). As it relates to couple engagement, a couple’s shared leisure activities play an important role in helping the couple develop continuity and a common identity (Johnson et al., 2006). Finally, communication is often viewed as one of the most important mechanisms related not only to couple engagement (Lavner et al., 2016), but as a catalyst for the sustainability of relationships (Markman et al., 2010).

All three of the aforementioned indicators of couple engagement have been linked to greater sexual intimacy and higher relationship quality (Berg et al., 2001; Gulledge et al., 2003; Johnson et al., 2006; Sharaievska et al., 2013; Dobson et al., 2020). However, few studies have visited couple engagement as a possible explanatory mechanism (i.e., mediator) of economic distress on sexual intimacy within the context of remarital unions (Crapo et al., 2021). The following sections review the existing literature on couple engagement in remarriage and the associations between couple engagement and sexual intimacy from the perspective of our theoretical framework.

##### Household labor

Although limited, research on the sharing of household labor among remarried couples has shown mixed results. Earlier research suggested remarried couples tend to display less traditional and more egalitarian arrangements compared to first-order marriages when it comes to the division of household labor (Shelton and Daphne, 1993; South and Spitze, 1994; Sullivan, 1997). However, there is some evidence that remarried couples tend to value traditional gender roles, with women continuing to assume the majority of household labor responsibilities (Ophir, 2022), rather than performing household labor together.

Generally speaking, the ways in which household labor is shared (or not) may influence sexual intimacy. Previous research has found mixed results, with both traditional (Kornrich et al., 2013) and more egalitarian (Carlson et al., 2016) approaches to division of household labor being associated with increased sexual activity. Other work by Harris et al. (2022) found that a perceived unfairness in the balance of household labor and less sharing resulted in lower sexual desire for women toward their male partners. Theoretically, misalignment with the vocational dimension could have an impact on a couple’s ability to successfully negotiate how household labor will be shared. If too much burden is placed on one partner or the other, it could lead to more negative outcomes related to sexual intimacy (e.g., lack of desire to be sexually intimate with a spouse due to feelings of exhaustion).

##### Leisure activities

Research on the prevalence of leisure or recreational activity patterns among remarried couples and stepfamilies is limited. Nicholas (2005) found no correlation between the amount of time stepfamilies spent engaged in leisure activities and their perceived levels of cohesion; however, the types of activities stepfamilies engaged in positively impacted their flexibility and emotional bonding. Some research on stepfamilies has speculated that remarried couples may have limited time for leisure or recreational activity due to child-related responsibilities, as well as limited economic resources due to child support or alimony obligations (Hafkin, 1981; DeLongis and Zwicker, 2017). Such an argument may further speak to the vocational, couple, and personal dimensions of MFDT, particularly if a lack of financial resources stemming from the vocational dimension limits opportunities for leisure activities, subsequently impeding the development of intimacy in the couple dimension (Crapo and Bradford, 2021).

Similar to arrangements related to the household division of labor, and as articulated by MFDT, meeting developmental needs within the couple dimension by engaging in leisure activities together holds many benefits for married couples. Spending meaningful and enjoyable time together (i.e., pairing) helps to nurture couple development, including in the area of sexual intimacy (Crapo and Bradford, 2021). Indeed, research on this topic indicates that when couples engage in satisfying leisure activities together, such activities can have positive effects on the quality of a couple’s romantic and sexual relationship (Dobson and Ogolsky, 2022), while also lowering the likelihood of relationship dissolution (Zabriskie and McCormick, 2001). Through leisure activities, couples not only enjoy greater relationship satisfaction, but they are able to develop shared interests and strengthen their communication skills (Berg et al., 2001; Johnson et al., 2006).

##### Communication

Research on the communication patterns of remarried couples has found that communication tends to be less positive when compared to couples in first-order marriages (Halford et al., 2007). Greater prevalence of negative communication may be associated with the unique challenges faced by remarried couples and stepfamilies (Hetherington and Stanley-Hagen, 1999) and the sensitive topics that may arise (Afifi and Schrodt, 2003), such as role ambiguity and relationships with ex-partners (Papernow, 2013; DeLongis and Zwicker, 2017), all of which create more opportunities for conflict.

Extensive research points to the importance of communication for the health and quality of couple relationships (Mark and Jozkowski, 2013; Hansson and Ahlborg, 2016), including in the areas of sexual intimacy and finances (Saxey et al., 2021; Wikle et al., 2021). Relative to sexual intimacy, research shows that frequent and positive communication play a pivotal role in nurturing satisfying sexual relationships for couples (Leistner and Mark, 2020). Positive and affectionate communication has also been linked to more frequent sexual contact (Willoughby et al., 2014; Debrot et al., 2017; Schoenfeld et al., 2017). In terms of finances, research has shown that more positive financial communication can improve couple relationship quality (Zimmerman and Roberts, 2012), which may explain why prosocial communication has also been identified as a preventative measure against potential economic distress (Conger et al., 1999). Taken together, the findings from this research supports the arguments of MFDT and the role of couple engagement as a manifestation of the couple’s success at meeting the portion of the family developmental task related to the couple dimension in a way that influences future outcomes related to positive and healthy sexual behaviors.

### Current study

Research is needed relative to how economic distress among remarried couples impacts other relationship domains, particularly the frequency of sexual intimacy (Crapo et al., 2021) and whether any mediating factors are at play. Based on empirical relations highlighted above, we propose that the couple dimension of development is likely to be influenced by misalignment with the vocational dimension (as measured by economic distress), such that couples will be less likely to meet needs in the couple dimension (measured by couple engagement and relationship satisfaction) resulting in a less optimal trajectory (manifested by sexual initiation and rejection). Using longitudinal data from remarried couples over 3 years, we tested the effects of economic distress in the first year (i.e., T1) on husband and wife self and partner reports of initiation of sexual intimacy with their partner 2 years later (i.e., T3) through the indirect paths of couple engagement and relationship satisfaction in the second year (i.e., T2). We explore these theoretical relationships through the following hypothesis:

1. There is a direct relationship between economic distress and sexual intimacy among remarried couples, wherein higher distress leads to decreased sexual intimacy.
2. Economic distress leads to decreased couple engagement among remarried couples.
3. Economic distress leads to decreased relationship satisfaction among remarried couples.
4. Increased couple engagement or satisfaction increases the sexual intimacy among remarried couples.
5. Couple engagement and relationship satisfaction acts as indirect paths in helping to explain the relationship between economic distress and sexual intimacy among remarried couples.

## Materials and methods

### Procedures

Data were gathered from couples in the first year of their remarriage. Remarried couples were identified through the review of marriage licenses issued in 2006. Data were acquired from the Office of Vital Records and Statistics in a western state, and surveys were sent to all remarried couples in April 2007. A remarried couple was defined as one in which at least one partner had been married previously, a common criteria used in defining remarriages (Livingston, 2014). Couples that fit this criteria (*n* = 4,886), were mailed an invitation to participate. A follow-up survey was sent in 2008, and then again in 2009, yielding data on three time points. A total of 2,042 participants responded to the survey at T1, including 881 husband-wife couples. Another 222 wives and 58 husbands returned the survey without their spouse’s response. Of those individuals, 1,038 also responded to the T2 survey, representing 620 couples. Of those who returned the survey, missing data was ~3%. Full information maximum likelihood (FIML) was used to account for attrition, partial couple responses, and missing data, with exogenous variables having their parameters freely estimated. As a result, we were able to include all 1,161 represented couples, whether their spouse returned the survey or not.

### Participants

Participants consisted of remarried couples who were in their first year of remarriage at the time first wave data were collected. The average age of the wife was 39.1 years (*SD* = 13.8 years), with 14 years of education (*SD* = 2.0), and on average she was on her second marriage (*M* = 2.1, *SD* = 1.0). The average age of the husband was 42.5 years (*SD* = 14.8), with 14 years of education (*SD* = 2.1), and on average he was on his second marriage as well (*M* = 2.1, *SD* = 0.08). The majority of the sample was White (97%). The average household income was between $45,000 and $50,000 annually. The couples had an average of 1.1 children living at home (*SD* = 1.4), with the wives reporting their oldest child as 20.6 years old (*SD* = 13.0 years) and husbands reporting their oldest child as 22.9 years old (*SD* = 14.6).

### Measures

#### Independent variable

Economic distress was measured at T1 using measures of felt economic constraint (Conger and Elder, 1994). Five items were measured on a scale of 1 (*strongly disagree*) to 5 (*strongly agree*). Items were summed to create a sum score. Example items included “*I often worry about my poor financial situation*” and “*My financial situation is much worse this year than it was last year*.” Cronbach’s alpha demonstrated sufficient internal consistency, with an alpha of 0.87 for men and 0.88 for women. Economic distress was modeled as a latent variable, with standardized loadings ranging from 0.63 to 0.86 for husbands and 0.64 to 0.87 for wives.

#### Mediators

Relationship satisfaction was measured at T2 using a two-item measure (Conger et al., 1990). Items were measured on a scale of 1 (*extremely unhappy*) to 7 (*extremely happy*). The two items were “*How happy are you with your marriage?*” and “*How satisfied are you with your relationship with your spouse?*” Items were summed to create a sum score. The two items were sufficiently correlated, with an *r* of 0.90 for men and 0.91 for women.

Couple engagement was measured at T2 through three variables: (1) household chores, (2) leisure activities, and (3) communication (Huston et al., 1986). Items were measured based on the average amount of time in hours and minutes that couples reported in engaging in household activities (e.g., eating meals or doing chores together), leisure activities (e.g., playing games or going to the movies together), and conversation together on a daily basis. Items were summed to create a couple engagement score. Cronbach’s alpha demonstrated sufficient internal consistency, with an alpha of 0.84 for men and 0.79 for women. Couple engagement was modeled as a latent variable, with standardized loadings ranging from 0.74 to 0.89 for husbands and 0.77 to 0.78 for wives.

#### Dependent variables

This study included four dependent variables. Frequency of the participant’s sexual initiation (*“In a typical day, how frequently do you initiate sex with your spouse?”*) and frequency of the participant’s rejection of their partner’s sexual advances (*“In a typical day, how frequently do you turn down or avoid sexual advances from your spouse?”*) were measured using one item from the socioemotional behavior index (SBI; Huston and Vangelisti, 1991), both of which were rated on a scale of 1 (*never*) to 5 (*always*). Perceptions of the frequency of the partner’s sexual initiation (*“In a typical day, how frequently does your spouse initiate sex with you?”*) and perceptions of the frequency of the partner’s rejection of sexual advances (*“In a typical day, how frequently does your spouse turn down or avoid sexual advances from you?”*) were also measured using one item from SBI and rated on a scale of 1 (*never*) to 5 (*always*).

#### Covariates (control variables)

Years of education, age, number of marriages, number of children living the in home, age of oldest child, and household income all served as covariates. With the exception of household income, all covariates were continuous variables. Household income was treated as an ordinal variable, with values ranging from less than $10,000 to more than $100,000 annually. These variables were selected as covariates due to their common usage in studies that focus on the functioning of remarriages and stepfamilies (Jensen and Shafer, 2013; Jensen et al., 2014; Crapo et al., 2021; Turner et al., 2021; Crapo et al., 2022).

### Plan of analysis

To answer our research questions, a dyadic structural equation model with indirect paths was employed. We regressed husbands’ self-reported initiation of sexual intimacy, self-reported rejection of their wives’ initiation, their report on wives’ frequency of initiation, and their report of their wives’ rejections of their initiation at T3 on husbands’ reports of T2 couple engagement and relationship satisfaction, and T1 reports of economic distress. Couple engagement and relationship satisfaction at T2 were also regressed on T1 reports of economic distress. Variables at T2 and T3 were correlated, respectively. In the same model, wives’ reports were regressed and correlated in the same fashion.

**Figure 2:**
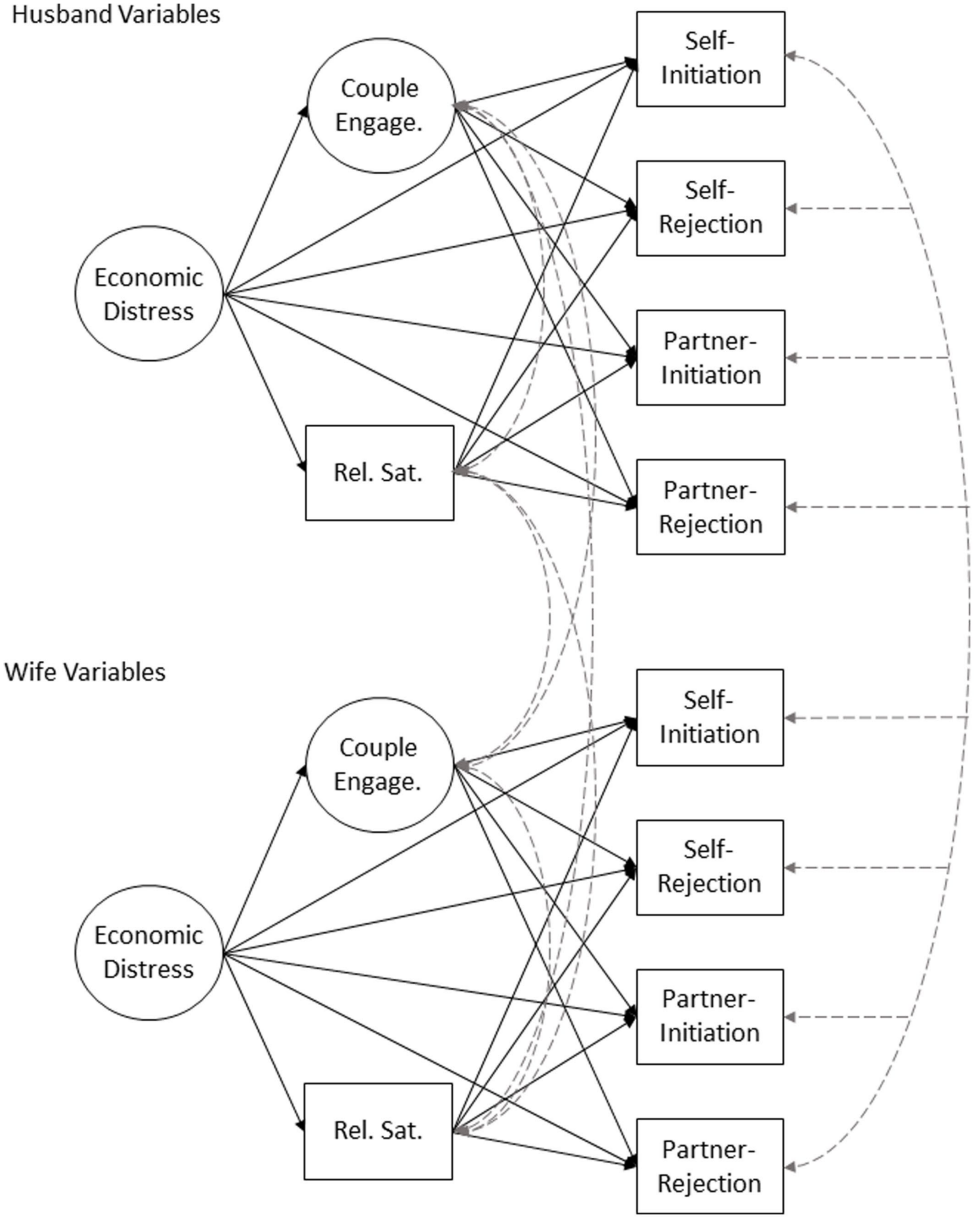
Specified model. Correlations are shown as dotted lines. For ease of reading, co-variates are not shown. “Self-” indicates reports on self-behavior, and “partner-” indicates reports on partner behavior.

To account for dependence of data, husband and wife outcomes (and intervening variables) were allowed to covary across spouse. See Figure 2 for a visual representation of the specified model. A nested version of the model where husband and wife paths were constrained to be equal was tested against the freely estimated version using a chi-square difference test to evaluate if husband and wife processes (i.e., regression paths) were the same or different. Covariates were then added to the chosen model by regressing outcomes onto the covariates. For the sake of parsimony, non-significant covariate regression paths were trimmed from the model. Model fit was evaluated using a non-significant chi-square test. Due to the sample size, in the case of a significant chi-square, we also considered goodness-of-fit indices. We considered a CFI ≥ 0.95, RMSEA ≤ 0.05 and SRMR ≤ 0.05 to constitute acceptable model fit (Hu and Bentler, 1999). Indirect paths were tested using a 95% CI with 5,000 bootstrapped samples. Analyses were conducted in R Core Team (2021), using the package lavaan (Rosseel, 2012).

## Results

**Figure 3:**
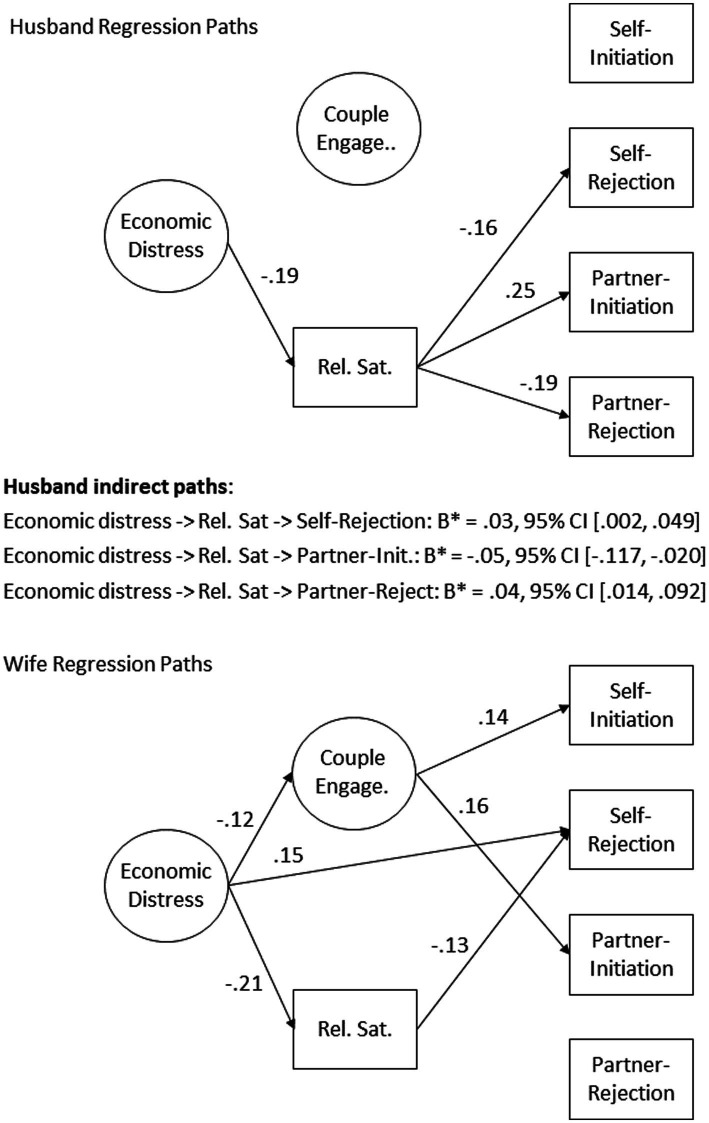
Estimated model (regression lines). Correlations and co-variates not shown. Standardized betas are reported for significant pathways. Non-significant paths not shown. 95% confidence intervals calculated using 5,000 bootstrapped samples. “Self-” indicates reports on self-behavior, and “partner-” indicates reports on partner behavior.

Means, *SD*, and correlations among study variables are given in Table 1. The version of the model where husband and wife paths were constrained to be equal demonstrated significantly worse fit, χ^2^(14) = 28.40, *p* = 0.013, leading us to reject that version and accept the version where husband and wife paths were freely estimated—implying the presence of gender effects. That is to say, the influence of economic distress, couple engagement, and relationship satisfaction on reports of sexual initiation and rejection differed between husband and wife. Removal of non-significant covariates did not substantively change parameter estimates. Number of children in the home and age of the oldest child were completely removed from the model, while the other covariates varied for which outcomes they were retained. Full details regarding retained covariates and regression paths of the final model are given in Table 2. The final model showed acceptable fit, χ^2^(361) = 784.24, *p* < 0.001, CFI = 0.95, RMSEA = 0.03, 95% CI [0.03, 0.04], SRMR = 0.05. The visual representation in Figure 3 displays the model’s significant regression paths, reporting standardized coefficients.

**Table 1:** Bivariate correlations, M, and SD among the observed variables.

		1	2	3	4	5	6	7	8	9	10	11	12	13	14	15	16	17	*M*	*SD*
1.	H. economic distress	--																	9.3	4.5
2.	W. economic distress	**0.51**	--																9.4	4.8
3.	H. couple engagement	0.09	−0.05	--															8.0	6.8
4.	W. couple engagement	0.06	−0.07	**0.67**	--														8.2	7.2
5.	H. relationship Sat.	**−0.22**	**−0.20**	**0.25**	**0.16**	--													12.4	2.0
6.	W. relationship Sat.	−0.12	**−0.22**	**0.14**	**0.17**	**0.51**	--												12.0	2.4
7.	H. self-initiation	−0.12	−0.12	−0.04	−0.02	0.11	0.07	--											2.8	1.2
8.	H. self-rejection	−0.03	−0.05	0.10	0.13	−0.07	**−0.15**	−0.09	--										1.3	0.6
9.	H. partner-initiation	**−0.20**	−0.12	0.06	0.00	**0.27**	0.11	**0.35**	0.03	--									2.4	1.1
10.	H. partner-rejection	**0.16**	**0.14**	−0.04	0.02	**−0.27**	**−0.16**	0.12	−0.08	**−0.47**	--								1.8	1.0
11.	W. self-initiation	0.02	−0.10	0.11	0.13	−0.08	0.01	0.00	0.08	**0.35**	**−0.18**	--							2.5	1.0
12.	W. self-rejection	0.13	**0.15**	−0.09	−0.10	**−0.14**	−0.11	0.06	−0.10	**−0.36**	**0.51**	**−0.21**	--						1.6	0.8
13.	W. partner-initiation	−0.03	−0.04	0.07	0.16	0.02	0.07	0.13	**−0.17**	0.08	**0.15**	**0.41**	**0.15**	--					3.0	1.2
14.	W. partner-rejection	0.04	**0.15**	−0.08	−0.06	−0.10	**−0.16**	−0.05	**0.35**	−0.04	0.00	0.03	−0.02	**−0.20**	--				1.4	0.7
15.	H. education	−0.11	**−0.22**	−0.06	0.00	−0.05	0.12	−0.01	0.09	−0.04	−0.08	0.10	0.00	0.00	−0.09	--			14.0	2.1
16.	Household income	**−0.46**	**−0.45**	**−0.17**	−0.12	0.01	0.13	0.05	0.10	0.04	**−0.15**	0.04	−0.09	−0.03	−0.04	**0.25**	--		16.2	4.4
17.	H. age	**−0.17**	**−0.16**	**0.14**	**0.18**	**0.14**	0.09	−0.03	0.07	**0.21**	**−0.41**	0.09	**−0.27**	**−0.15**	0.01	0.11	0.06	--	42.5	14.8
18.	W. age	**−0.16**	−0.13	0.11	**0.15**	**0.14**	0.07	−0.05	0.05	**0.22**	**−0.40**	0.10	**−0.26**	−0.12	0.02	0.06	0.04	**0.90**	39.1	13.8

H is the husband report and W is the wife report. “self-” refers to report on self, and “partner-” refers to report on partner. M and SD for Economic Distress and Couple Engagement is calculated from the sum score. Bolded values are significant at the *p* < 0.05 level.

**Table 2:** Regressions from the final model.

Husband				Wife			
Predictor	B	*p*	B*	Predictor	B	*p*	B*
Self-initiation				Self-initiation			
Economic distress	−0.10	0.228	−0.07	Economic distress	−0.10	0.106	−0.08
Couple engage.	−0.01	0.739	−0.02	Couple engage.	0.07	**0.015**	0.14
Relationship sat.	0.06	0.081	0.10	Relationship sat.	0.02	0.440	0.04
Self-rejection				Self-rejection			
Economic distress	0.02	0.642	0.02	Economic distress	0.11	**0.007**	0.14
Couple engage.	0.03	0.073	0.11	Couple engage.	0.01	0.768	0.02
Relationship sat.	−0.05	**0.005**	−0.16	Relationship sat.	−0.04	**0.021**	−0.13
Education	0.03	**0.017**	0.11	Wife age	−0.01	**0.004**	−0.12
Partner-initiation				Partner-initiation			
Economic distress	−0.10	0.179	−0.07	Economic distress	0.02	0.820	0.01
Couple engage.	0.00	0.936	0.01	Couple engage.	0.09	**0.007**	0.16
Relationship sat.	0.14	**0.000**	0.25	Relationship sat.	0.04	0.110	0.09
Husband age	0.01	**0.013**	0.11	Partner-rejection			
Marriage num.	−0.11	0.054	−0.08	Economic distress	0.05	0.208	0.07
Partner-rejection				Couple engage.	−0.02	0.183	−0.08
Economic distress	0.01	0.876	0.01	Relationship sat.	−0.02	0.175	−0.08
Couple engage.	0.04	0.183	0.08	Couple engagement			
Relationship sat.	−0.10	**0.000**	−0.19	Economic distress	−0.28	**0.024**	−0.12
Husband age	−0.02	**0.000**	−0.28	Wife age	0.03	**0.000**	0.16
Couple engagement				Household income	−0.12	**0.001**	−0.24
Economic distress	−0.12	0.440	−0.04	Relationship sat.			
Husband age	0.03	**0.000**	0.19	Economic distress	−0.64	**0.000**	−0.24
Education	−0.11	**0.029**	−0.10	Economic distress			
Household income	−0.11	**0.000**	−0.22	Household income	−0.09	**0.000**	−0.44
Relationship sat.				Husband Age	−0.01	0.129	−0.13
Economic distress	−0.45	**0.000**	−0.19	Wife Age	−0.00	0.378	−0.06
Economic distress							
Household income	−0.08	**0.000**	−0.44				
Husband age	0.00	0.447	0.05				
Wife age	−0.02	**0.000**	−0.28				

Indented variables were predictors of the non-indented variables above them. “Self-” indicates reports on self-behavior, and “partner-” indicates reports on partner behavior. Bolded values denote significant correlations at the *p* < 0.05 level.

### Husbands

For husbands, report of self-initiation of sexual intimacy at T3 was not predicted by economic distress at T1, nor was it predicted by couple engagement or relationship satisfaction at T2. Husband relationship satisfaction at T2 was predictive of his rejection of his wife’s sexual initiation (B* = −0.16, *p* = 0.005), his wife’s initiation (B* = 0.25, *p* < 0.001), and his wife’s rejection of his initiation at T3 (B* = −0.19, *p* < 0.001); these variables were not predicted by couple engagement at T2 or economic distress at T1. Economic distress at T1 did have a small, but significant, indirect effect through relationship satisfaction at T2 on T3 husbands’ reports of his rejection of his wife’s sexual initiation (B* = 0.03, 95% CI [0.002, 0.049]), his wife’s initiation (B* = −0.0595% CI [−0.117, −0.020]), and his wife’s rejection of his initiation (B* = 0.04, 95% CI [0.014, 0.092]) at T3.

### Wives

Wives’ reports of the rejection of their husband’s sexual advances at T3 were directly predicted by economic distress at T1 (B* = 0.14, *p* = 0.007), and relationship satisfaction at T2 (B* = −0.13, *p* = 0.021). Next, wives’ reports of her initiation and her partner’s initiation at T3 were directly predicted by her couple engagement at T2 (B* = 0.14, *p* = 0.015, B* = 0.16, *p* = 0.007, respectively). Wives’ reports of their husbands’ rejection of their initiation was not predicted by any of the analyzed variables. The model for wives demonstrated no indirect paths.

## Discussion

Grounded in MFDT, this study examined the relationships between economic distress, couple engagement, relationship satisfaction, and perceptions of sexual intimacy among remarried couples. More specifically, longitudinal data from remarried couples were analyzed to explore the impact of economic distress on sexual intimacy among remarried couples, the relationship between economic distress and couple engagement, and the relationship between economic distress and relationship satisfaction. Further, we tested the impact of couple engagement and satisfaction on the sexual intimacy of remarried couples and whether couple engagement and relationship satisfaction acted as indirect factors in helping to explain the relationship between economic distress and sexual intimacy. Although estimated in the same model, we discuss the unique effects for husbands and wives. This is because the model where husband and wife paths were constrained to be equal demonstrated significantly worse fit, implying the presence of gender effects. Given the lack of research on this topic in the context of remarriage, we applied key elements from multidimensional family development theory (MFDT; Crapo and Bradford, 2021). The following sections present an evaluation of this study’s significant findings.

### Economic distress and physical intimacy

Higher levels of economic distress among wives resulted in more frequent rejection of her husband’s sexual advances. However, this was not found to be the case for husbands. These findings are consistent with the work of Saxey et al. (2021) and Wikle et al. (2021), which also found significant links between the economic distress felt by wives and their sexual outcomes. Although speculative, the direct relationship between a wife’s economic distress and her rejecting a husband’s sexual advances in the context of remarriage, may be related to the economic strain resulting from the husband’s financial obligations outside the home in the form of alimony or child support. Indeed, past research has shown that this can cause tension in remarriages (Sweeney, 2010). Furthermore, evolutionary psychology posits that women place greater emphasis on financial security when seeking a mate (Silveri and Samayoa, 2018). When this security is threatened, their sexual intimacy appears to be compromised. From the perspective of our theoretical framework, this finding confirms that strained resources in the vocational dimension may lead to greater levels of economic distress, a form of misalignment which impacts the dimension of couple development, notably within the realm of sexual intimacy (Crapo and Bradford, 2021).

### Economic distress and couple engagement

Results indicated that, for wives, as economic distress increased, couple engagement decreased. The significant findings between economic distress and couple engagement for wives indicate that greater economic distress had a perceived impact on the amount of time wives felt their husbands (or they as a couple) spent engaging in tasks related to couple engagement. Such findings are consistent with past research on remarried couples, especially as it relates to leisure activities, where economic distress brought on by limited economic resources may in turn limit time and financial means to engage in leisure activities (Hafkin, 1981; DeLongis and Zwicker, 2017).

### Economic distress and relationship satisfaction

For both husbands and wives, economic distress negatively impacted relationship satisfaction, such that when spouses reported economic distress, they were less satisfied with their relationship, which is consistent with past research (Dew and Dakin, 2011). From a MFDT standpoint, it could be argued that these findings are evidence of how the strain of economic distress, possibly brought on by the vocational dimension, impede healthy development in the couple dimension (Crapo and Bradford, 2021). Couples might benefit from resources (e.g., interventions) that would teach them effective money management strategies (Li et al., 2021).

### Couple engagement, satisfaction, and physical intimacy

Perhaps the most striking differences between husbands and wives were the divergent roles that couple engagement and satisfaction played in terms of a remarried couple’s sexual intimacy. For husbands, couple engagement showed no impact on any of the measures of sexual intimacy. However, greater relationship satisfaction among husbands was predictive of less rejection of his partner’s advances, more perceived sexual initiation from his partner, and less perceived rejection from his partner of his sexual advances. Such findings suggest that relationally satisfied husbands are prone to show their affection through sexual intimacy, which is consistent with past research (Butzer and Campbell, 2008; Smith et al., 2011).

Conversely, couple engagement was more important for wives in terms of self and partner initiated sexual intimacy. In other words, from the wives’ perspectives, more time spent engaging in activities together (i.e., household labor, leisure activities, or conversation) lead to greater frequency of their own and their partner’s initiation of sexual intimacy. These differences may help to demonstrate the priority that husbands and wives place on different elements of a relationship, how these priorities differ by gender, and how this ultimately affects the sexual intimacy of remarried couples.

Generally, research has shown that husbands often show higher levels of relationship satisfaction than wives (Jackson et al., 2014). In the current study, it appears that a husband’s positive perceptions of the overall relationship were linked to positive perceptions of the sexual intimacy aspects of the relationship. The fact that wives reported initiating sexual intimacy more frequently and also perceived that their husbands initiated sexual intimacy more frequently when more time was spent engaging in household labors, leisure activities, and conversation may indicate that a more shared time in household labor (which may be reflective of greater equity in the division of labor) and higher satisfaction with leisure activities and communication could lead to greater frequency of sexual intimacy for remarried couples. Such a finding supports the MFDT framework, especially as it relates to how the resolution of family developmental tasks can help to foster healthy development within the couple dimension (Crapo and Bradford, 2021).

### Mediating effects of couple engagement and satisfaction

For husbands, economic distress did not have a direct effect on any of the measures of sexual intimacy; however, with the exception of self-reported initiation of sexual intimacy, economic distress had an indirect effect through relationship satisfaction on all of the husbands’ reports of sexual frequency. Previous research has found a connection between early economic distress and husband sexual intimacy (Crapo et al., 2021). This suggests that any effect of economic distress is fully mediated by relationship satisfaction. As such, interventions targeted toward minimizing the impact of economic distress on satisfaction (such as through improved financial communication or financial therapy; Zimmerman and Roberts, 2012; Wikle et al., 2021) may be a mechanism by which to help stabilize and improve remarriages from the husband’s perspective.

No indirect effects were found for wives, only a direct effect. This suggests that although economic distress influences both her perception of engagement and rejection of her husband’s sexual advances, engagement is not the mechanism by which economic distress influences sexual intimacy for wives in remarriage. Rather, there appears to be something about the economic distress itself, or other unknown variables. The indirect role of relationship satisfaction from economic distress to husbands’ reports of sexual frequency, and the direct effect of couple engagement on wives’ reports of sexual frequency, provides empirical clarity on the interdependence between emotional and sexual intimacy within the couple dimension (Crapo and Bradford, 2021).

### Strengths, limitations, and future directions

The main strength of this study was its focus on how economic distress and other relationship aspects (i.e., couple engagement and satisfaction) impact the sexual intimacy of remarried couples through the analysis of dyadic, longitudinal data. This is one of few studies on this topic (see Crapo et al., 2021). The findings of this study may inspire further investigation into the unique challenges faced by remarried couples and stepfamilies, particularly in the area of sexual intimacy.

Limitations of the current study could help to inform future research. For example, we relied on self-reported data, which may differ from observed behavior. Our findings also may not generalize to first-order marriages or cohabitating couples. Next, our sample also lacked racial and ethnic diversity, as the majority of participants were White and they all resided in one state. Future research would be well-served to seek out more diverse samples residing in different areas. Our measures included a two-item and single-item measures, which are considered less psychometrically robust than longer, validated measures. Finally, exploring how parenting (or stepparenting) challenges influence sexual intimacy in remarriage could also be a possibility for future research. Indeed, the presence of children and stepfamily formation has been found to add another dynamic to the transitions associated with remarriage (Gold, 2016).

### Implications

This study holds implications for both researchers and practitioners alike. From a research perspective, this study is one of the few to look at the predictors of sexual intimacy among remarried couples (Crapo et al., 2021), opening up further possibilities for research on this and related topics. Further, the application of multidimensional family development theory (MFDT; Crapo and Bradford, 2021) supports the utility and potential of this emerging theory in helping explain the relationship dynamics of remarried couples and stepfamilies, especially within the context of their developmental history. Indeed, several issues surrounding remarriage and stepfamily formation are unique when compared to first-order marriages, especially as it relates to aligning the resources necessary to achieve the developmental tasks of the family unit, something MFDT is well situated to help explain.

Our findings suggest that economic distress among wives may increase their rejection of a partner’s sexual advances. Relationship satisfaction played more of a role for husbands, while couple engagement acted as a significant predictor for wives. These findings may guide practitioners attempting to help remarried couples. That is, it may be important for the practitioner seeking to improve a remarried wife’s sexual intimacy to focus on helping the couple to engage in more activities together, or helping a husband to increase his satisfaction. Because economic distress directly impacted wives’ sexual perceptions, it may be important to help both spouses understand the role of economic distress.

Economic distress is a common issue that remarried couples face (Ganong and Coleman, 2017). Helping couples understand the impact of misalignment, in this case due to economic distress, can have on other aspects of their relationship can assist them in identifying possible warning signs. The use of MFDT can also provide additional avenues of help. By helping the couple identify which needs in the relationship are impacted by economic distress, the practitioner could help them to take preventative measures and meet developmental needs within the personal and couple dimension. Certainly, focusing on the sexual intimacy of remarried couples illustrates the importance of finding a way to nurture their marital relationship and achieve couple cohesiveness.

## Conclusion

The current study adds to the limited body of work on the role economic distress can play on the perceptions of sexual intimacy among remarried couples. This study also points to the gender differences that may exist between remarried husbands and wives, especially considering the contrast in terms of how couple engagement and relationship satisfaction play different roles in influencing the perceptions of frequency of sexual intimacy for the respective groups. From the perspective of MFDT, our findings support the further application of this theory, especially as it relates to the couple dimension of development for remarried couples. Finally, this study highlights potential benefits for practitioners developing interventions for remarried couples and stepfamilies.

## Data availability statement

The original contributions presented in the study are included in the article/supplementary material, further inquiries can be directed to the corresponding authors.

## Author contributions

OK, KB, and BH contributed to the conceptualization, research design, and editing of the manuscript. All authors contributed to the article and approved the submitted version.

## Conflict of interest

The authors declare that the research was conducted in the absence of any commercial or financial relationships that could be construed as a potential conflict of interest.

The reviewer AL-B declared a past co-authorship with the author OK to the handling editor.

## Publisher’s note

All claims expressed in this article are solely those of the authors and do not necessarily represent those of their affiliated organizations, or those of the publisher, the editors and the reviewers. Any product that may be evaluated in this article, or claim that may be made by its manufacturer, is not guaranteed or endorsed by the publisher.

## References

[ref1] Afifi J. D. Schrodt P. (2003). Uncertainty and the avoidance of the state of one’s family in stepfamilies, post-divorce single parent families, and first marriage families. Hum. Commun. Res. 29, 516–532. doi: 10.1111/j.1468-2958.2003.tb00854.x

[ref2] Berg E. C. Trost M. Schneider I. E. Allison M. T. (2001). Dyadic exploration of the relationship of leisure satisfaction, leisure time, and gender to relationship satisfaction. Leis. Sci. 23, 35–46. doi: 10.1080/01490400150502234

[ref3] Britt S. L. Huston S. J. (2012). The role of money arguments in marriage. J. Fam. Econ. Iss. 33, 464–476. doi: 10.1007/s10834-012-9304-5

[ref4] Buck A. A. Neff L. A. (2012). Stress spillover in early marriage: the role of self-regulatory depletion. J. Fam. Psychol. 26, 698–708. doi: 10.1037/a0029260, PMID: 22866931

[ref5] Butzer B. Campbell L. (2008). Adult attachment, sexual satisfaction, and rela-tionship satisfaction: a study of married couples. Pers. Relat. 15, 141–154. doi: 10.1111/j.1475-6811.2007.00189.x

[ref6] Cao H. Zhou N. Fine M. A. Li X. Fang X. (2019). Sexual satisfaction and marital satisfaction during the early years of Chinese marriage: a three-wave, cross-lagged, actor–partner interdependence model. J. Sex Res. 56, 391–407. doi: 10.1080/00224499.2018.1463503, PMID: 29746180

[ref7] Carlson D. L. Miller A. J. Sassler S. Hanson S. (2016). The gendered division of housework and couples’ sexual relationships: a reexamination. J. Marriage Fam. 78, 975–995. doi: 10.1111/jomf.12313

[ref8] Ciciolla L. Luthar S. S. (2019). Invisible household labor and ramifications for adjustment: mothers as captains of households. Sex Roles 81, 467–486. doi: 10.1007/s11199-018-1001-x, PMID: 34177072 PMC8223758

[ref9] Conger R. D. Elder G. H. (1994). Families in troubled times: Adapting to change in rural America. New York: Walter de Gruyter.

[ref10] Conger R. D. Elder G. H. Lorenz F. O. Conger K. J. Simons R. L. Whitbeck L. B.. (1990). Linking economic hardship to marital quality and instability. J. Marriage Fam. 52, 643–656. doi: 10.2307/352931

[ref11] Conger R. D. Rueter M. A. Elder G. H. Jr. (1999). Couple resilience to economic pressure. J. Pers. Soc. Psychol. 76, 54–71. doi: 10.1037/0022-3514.76.1.54, PMID: 9972553

[ref12] Copen C. E. Daniels K. Vespa J. Mosher W. D. (2012). First marriage in the United States: Data from the 2006–2010 national survey of family growth. Atlanta: U.S. Department of Health and Human Services. 22803221

[ref13] Crapo J. S. Bradford K. (2021). Multidimensional family development theory: a reconceptualization of family development. J. Fam. Theory Rev. 13, 202–223. doi: 10.1111/jftr.12414

[ref14] Crapo J. S. Turner J. J. Bradford K. Higginbotham B. J. (2022). The impacts of post-divorce cohabitation and relationship duration on the early marital climate of remarriages. Fam. J. 30, 307–315. doi: 10.1177/10664807211054155

[ref15] Crapo J. S. Turner J. J. Kopystynska O. Bradford K. Higginbotham B. J. (2021). Financial stress and perceptions of spousal behavior over time in remarriage. J. Fam. Econ. Iss. 42, 300–313. doi: 10.1007/s10834-020-09697-6

[ref16] Debrot A. Meuwly N. Muise A. Impett E. A. Schoebi D. (2017). More than just sex: affection mediates the association between sexual activity and well-being. Pers. Soc. Psychol. Bull. 43, 287–299. doi: 10.1177/0146167216684124 28903688

[ref17] DeLongis A. Zwicker A. (2017). Marital satisfaction and divorce in couples in stepfamilies. Curr. Opin. Psychol. 13, 158–161. doi: 10.1016/j.copsyc.2016.11.003, PMID: 28813287

[ref18] Dew J. Britt D. Huston S. (2012). Examining the relationship between financial issues and divorce. Fam. Relat. 61, 615–628. doi: 10.1111/j.1741-3729.2012.00715.x

[ref19] Dew J. P. Dakin J. (2011). Financial disagreements and marital conflict tactics. J. Fin. Ther. 2, 23–42. doi: 10.4148/jft.v2i1.1414

[ref20] Dobson K. Ogolsky B. (2022). The role of social context in the association between leisure activities and romantic relationship quality. J. Soc. Pers. Relat. 39, 221–244. doi: 10.1177/02654075211036504

[ref21] Dobson K. Zhu J. Balzarini R. N. Campbell L. (2020). Responses to sexual advances and satisfaction in romantic relationships: is yes good and no bad? Soc. Psychol. Personal. Sci. 11, 801–811. doi: 10.1177/1948550619888884

[ref22] Falke S. L. Larson J. H. (2007). Premarital predictors of remarital quality: implications for clinicians. Contemp. Fam. Ther. 29, 9–23. doi: 10.1007/s10591-007-9024-4

[ref23] Ganong L. Coleman M. (2017). Stepfamily relationships: Development, dynamics, and interventions (2nd ed.). New York: Springer.

[ref24] Gold J. M. (2016). Stepping in, stepping out: Creating stepfamily rhythm. Wiley, Alexandria, VA: American Counseling Association.

[ref25] Gulledge A. K. Gulledge M. H. Stahmannn R. F. (2003). Romantic physical affection types and relationship satisfaction. Am. J. Fam. Ther. 31, 233–242. doi: 10.1080/01926180390201936

[ref26] Hafkin N. F. (1981). Factor affecting satisfaction in the stepfamily couple (publication no. 8124269). Doctoral dissertation. The American University. ProQuest Dissertations Publishing.

[ref27] Halford K. Nicholson J. Sanders M. (2007). Couple communication in stepfamilies. Fam. Process 46, 471–483. doi: 10.1111/j.1545-5300.2007.00226.x 18092580

[ref28] Hansson M. Ahlborg T. (2016). Factors contributing to separation/divorce in parents of small children in Sweden. Nordic Psychol. 68, 40–57. doi: 10.1080/19012276.2015.1071201

[ref29] Harris E. A. Gormezano A. M. van Anders S. M. (2022). Gender inequities in household labor predict lower sexual desire in women partnered with men. Arch. Sex. Behav. 51, 3847–3870. doi: 10.1007/s10508-022-02397-2, PMID: 36112330 PMC9483460

[ref30] Henderson-King D. H. Veroff J. (1994). Sexual satisfaction and marital well-being in the first years of marriages. J. Soc. Pers. Relat. 11, 509–534. doi: 10.1177/0265407594114002

[ref31] Hetherington M. Stanley-Hagen M. (1999). The adjustment of children with divorced parents: a risk and resiliency perspective. J. Child Psychol. Psychiatry Allied Discip. 40, 129–140. doi: 10.1111/1469-7610.00427, PMID: 10102729

[ref32] Hill E. J. Allsop D. B. LeBaron A. B. Bean R. A. (2017). How do money, sex, and stress influence marital instability? J. Fin. Ther. 8, 21–42. doi: 10.4148/1944-9771.1135

[ref33] Hu L. T. Bentler P. M. (1999). Cutoff criteria for fit indexes in covariance structure analysis: conventional criteria versus new alternatives. Struct. Equ. Modeling 6, 1–55. doi: 10.1080/10705519909540118

[ref34] Huston T. L. McHale S. Crouter A. C. (1986). “When the honeymoon’s over: changes in the marriage relationship over the first year” in The emerging field of personal relationships. eds. Gilmour R. Duck S. (London and New York: Routledge), 109–131.

[ref35] Huston T. L. Vangelisti A. L. (1991). Socioemotional behavior and satisfaction in marital relationships: a longitudinal study. J. Pers. Soc. Psychol. 61, 721–733. doi: 10.1037/0022-3514.61.5.721, PMID: 1753328

[ref36] Jackson J. B. Miller R. B. Oka M. Henry R. G. (2014). Gender differences in marital satisfaction; a meta-analysis. J. Marriage Fam. 76, 105–129. doi: 10.1111/jomf.12077

[ref37] Jenkins N. H. Stanley S. M. Bailey W. C. Markman H. J. (2002). You paid how much for that? How to win at money without losing at love. San Francisco, CA: Jossey-Bass.

[ref38] Jensen T. M. Shafer K. (2013). Stepfamily functioning and closeness: Children’s views on second marriages and stepfather relationships. Soc. Work 58, 127–136. doi: 10.1093/sw/swt007, PMID: 23724576

[ref39] Jensen T. M. Shafer K. Larson J. H. (2014). (step)parenting attitudes and expectations: implications for stepfamily functioning and clinical intervention. Fam. Soc. 95, 213–220. doi: 10.1606/1044-3894.2014.95.27

[ref40] Johnson H. A. Zabriskie R. B. Hill B. (2006). The contribution of couple leisure involvement, leisure time, and leisure satisfaction to marital satisfaction. Marriage Fam. Rev. 40, 69–91. doi: 10.1300/J002v40n01_05

[ref41] Kapelle N. (2022). Time cannot heal all wounds: wealth trajectories of divorcees and the married. J. Marriage Fam. 84, 592–611. doi: 10.1111/jomf.12824, PMID: 35874926 PMC9303434

[ref42] Kapelle N. Baxter J. (2021). Marital dissolution and personal wealth: examining gendered trends across the dissolution process. J. Marriage Fam. 83, 243–259. doi: 10.1111/jomf.12707

[ref43] Kelley H. H. LeBaron-Black A. Hill J. E. (2018). Financial stress and marital quality: the moderating influence of couple communication. J. Fin. Ther. 9, 18–36. doi: 10.4148/1944-9771.1176

[ref44] Kim J. Muise A. Impett E. A. (2018). The relationship implications of rejecting a partner for sex kindly versus having sex reluctantly. J. Soc. Pers. Relat. 35, 485–508. doi: 10.1177/0265407517743084

[ref45] Kornrich S. Brines J. Leupp K. (2013). Egalitarianism, housework, and sexual frequency in marriage. Am. Sociol. Rev. 78, 26–50. doi: 10.1177/0003122412472340, PMID: 25540459 PMC4273893

[ref46] Lavner J. A. Karney B. R. Bradbury T. N. (2016). Does couples’ communication predict marital satisfaction, or does marital satisfaction predict communication? J. Marriage Fam. 78, 680–694. doi: 10.1111/jomf.12301, PMID: 27152050 PMC4852543

[ref47] Leavitt C. E. Dew J. P. Allsop D. B. Runyan S. D. Hill E. J. (2019). Relational and sexual costs of materialism in couple relationships: an actor-partner longitudinal study. J. Fam. Econ. Iss. 40, 438–454. doi: 10.1007/s10834-019-09617-3

[ref48] Leistner C. E. Mark K. P. (2020). Positive communication and partner appraisals among mothers and their long-term male partners: impact on sexual and relationship satisfaction. J. Sex Marital Ther. 46, 269–281. doi: 10.1080/0092623X.2019.1692980, PMID: 31777315

[ref49] Li X. Curran M. A. LeBaron-Black A. B. Jorgensen B. Yorgason J. Wilmarth M. J. (2021). Couple-level attachment styles, finances, and marital satisfaction: mediational analyses among young adult newlywed couples. J. Fam. Econ. Iss. doi: 10.1007/s10834-021-09808-x

[ref50] Livingston G. (2014). Four-in-ten couples are saying “I do,” again: Growing number of adults have remarried. Pew Research Center. Available at: https://www.pewsocialtrends.org/2014/11/14/four-in-ten-couples-are-saying-i-do-again/

[ref51] Mark K. P. Jozkowski K. N. (2013). The mediating role of sexual and nonsexual communication between relationship and sexual satisfaction in a sample of college-age heterosexual couples. J. Sex Marital Ther. 39, 410–427. doi: 10.1080/0092623X.2011.644652, PMID: 23530670

[ref52] Markman H. J. Rhoades G. K. Stanley S. M. Ragan E. P. Whitton S. W. (2010). The premarital communication roots of marital distress and divorce: the first five years of marriage. J. Fam. Psychol. 24, 289–298. doi: 10.1037/a0019481, PMID: 20545402 PMC4298140

[ref53] McNulty J. K. Wenner C. A. Fisher T. D. (2016). Longitudinal associations among relationship satisfaction, sexual satisfaction, and frequency of sex in early marriage. Arch. Sex. Behav. 45, 85–97. doi: 10.1007/s10508-014-0444-6, PMID: 25518817 PMC4472635

[ref54] Nicholas N. N. (2005). Family leisure and its relationship to cohesion in stepfamilies (publication no. 3179179). Doctoral dissertation. Capella University. ProQuest Dissertations Publishing.

[ref55] Olson D. H. DeFrain J. Skogrand L. (2021). Marriages and families: Intimacy, diversity, and strengths. 10th Edn. New York: McGraw-Hill.

[ref56] Ophir A. (2022). “Thank u, next”? Repartnering and the household division of labor. J. Marriage Fam. 84, 636–654. doi: 10.1111/jomf.12816, PMID: 35756753 PMC9231825

[ref57] Papernow P. (2013). Surviving and thriving in stepfamily relationships: What works and what doesn’t. New York, NY: Routledge.

[ref58] Patterson J. M. (2002). Integrating family resilience and family stress theory. J. Marriage Fam. 64, 349–360. doi: 10.1111/j.1741-3737.2002.00349.x

[ref59] R Core Team. (2021). R: A language and environment for statistical computing (version 3.6.3) [computer software]. Vienna, Austria: R Foundation for Statistical Computing.

[ref60] Rosseel Y. (2012). Lavaan: an R package for structural equation modeling. J. Stat. Softw. 48, 1–36. doi: 10.18637/jss.v048.i02

[ref61] Sassler S. (2010). Partnering across the life course: sex, relationships, and mate selection. J. Marriage Fam. 72, 557–575. doi: 10.1111/j.1741-3737.2010.00718.x, PMID: 22822268 PMC3399251

[ref62] Saxey M. T. Leavitt C. E. Dew J. P. Yorgason J. B. Holmes E. K. LeBaron-Black A. B. (2021). The budget and the bedroom: associations between financial management behaviors, perceptions of economic pressure, and sexual satisfaction. J. Fin. Ther. 12, 21–46. doi: 10.4148/1944-9771.1266

[ref63] Schoenfeld E. A. Loving T. J. Pope M. T. Huston T. L. Štulhofer A. (2017). Does sex really matter? Examining the connections between spouses’ nonsexual behaviors, sexual frequency, sexual satisfaction, and marital satisfaction. Arch. Sex. Behav. 46, 489–501. doi: 10.1007/s10508-015-0672-4, PMID: 26732606

[ref64] Sharaievska I. Kim J. Stodolska M. (2013). Leisure and marital satisfaction in intercultural marriages. J. Leis. Res. 45, 445–465. doi: 10.18666/jlr-2013-v45-i4-3894

[ref65] Shelton B. A. Daphne J. (1993). Does marital status make a difference? Housework among married and cohabitating men and women. J. Fam. Issues 14, 401–420. doi: 10.1177/019251393014003004

[ref66] Silveri A. Samayoa M. F. (2018). “Beyond the honeymoon: physical attraction and its role throughout marriage,” in The psychology of marriage: An evolutionary and cross-cultural view. eds. Weisfeld C. Weisfeld G. Dillon L. (Lanham, Maryland: Lexington Books), 211–212.

[ref67] Smith A. Lyons A. Ferris J. Richters J. Pitts M. Shelley J.. (2011). Sexual and relationship satisfaction among heterosexual men and women: the importance of desired frequency of sex. J. Sex Marital Ther. 37, 104–115. doi: 10.1080/0092623X.2011.560531, PMID: 21400335

[ref68] Snyder D. K. Heyman R. W. E. Haynes S. N. Carlson C. I. Balderrama-Durbin C. (2016). “Couple and family assessment,” in APA handbook of clinical psychology: Applications and methods (Vol. 3). eds. Norcross J. C. VandenBos G. B. Freedheim D. K. Vol. 3 (Washington, DC: American Psychological Association), 201–217.

[ref69] South S. J. Spitze G. (1994). Housework in marital and non-marital households. Am. Sociol. Rev. 59, 327–347. doi: 10.2307/2095937

[ref70] Sullivan O. (1997). The division of housework among “remarried” couples. J. Fam. Issues 18, 205–223. doi: 10.1177/019251397018002005

[ref71] Sweeney M. (2010). Remarriage and stepfamilies: strategic sites for family scholarship in the 21st century. J. Marriage Fam. 72, 667–684. doi: 10.1111/j.1741-3737.2010.00724.x

[ref72] Turner J. J. Kopystynska O. Bradford K. Schramm D. G. Higginbotham B. J. (2021). Predicting parenting and stepparenting difficulties among newly remarried parents. J. Divorce Remarriage 62, 511–531. doi: 10.1080/10502556.2021.1925857

[ref73] Vowels L. M. Mark K. P. (2020). Relationship and sexual satisfaction: a longitudinal actor–partner interdependence model approach. Sex. Relatsh. Ther. 35, 46–59. doi: 10.1080/14681994.2018.1441991

[ref74] Wheeler B. Kerpelman J. (2016). Change in disagreements about money, time, and sex and marital outcomes. J. Relationsh. Res. 7:e6. doi: 10.1017/jrr.2016.8

[ref75] Wikle J. S. Leavitt C. E. Yorgason J. B. Dew J. P. Johnson H. M. (2021). The protective role of couple communication in moderating negative associations between financial stress and sexual outcomes for newlyweds. J. Fam. Econ. Iss. 42, 282–299. doi: 10.1007/s10834-020-09728-2

[ref76] Willoughby B. J. Farero A. M. Busby D. M. (2014). Exploring the effects of sexual desire discrepancy among married couples. Arch. Sex. Behav. 43, 551–562. doi: 10.1007/s10508-013-0181-2, PMID: 24045904

[ref77] Yeh H. C. Lorenz F. O. Wickrama K. A. S. Conger R. D. Elder G. H. (2006). Relationships among sexual satisfaction, marital quality, and marital instability at midlife. J. Fam. Psychol. 20, 339–343. doi: 10.1037/0893-3200.20.2.339, PMID: 16756411

[ref78] Zabriskie R. B. McCormick B. P. (2001). The influences of family leisure patterns on perceptions of family functioning. Fam. Relat. 50, 281–289. doi: 10.1111/j.1741-3729.2001.00281.x

[ref79] Zimmerman K. J. Roberts C. W. (2012). The influence of a financial management course on couples’ relationship quality. J. Financ. Couns. Plan. 23, 46–54. doi: 10.31274/etd-180810-1386

